# Global research trends of hypertrophic cardiomyopathy from 2000 to 2022: Insights from bibliometric analysis

**DOI:** 10.3389/fcvm.2023.1039098

**Published:** 2023-02-02

**Authors:** Xifeng Zheng, Zhongkai He, Ming Li, Zhen Jia

**Affiliations:** ^1^Department of Cardiology, Affiliated Hospital of Guangdong Medical University, Zhanjiang, China; ^2^Department of Geriatrics, Affiliated Hospital of Guangdong Medical University, Zhanjiang, China

**Keywords:** hypertrophic cardiomyopathy, bibliometric analysis, Mavacamten, genetic diagnosis, cardiac magnetic resonance, web of science

## Abstract

**Objectives:**

To analyze the global research trends of hypertrophic cardiomyopathy (HCM) from 2000 to 2022 and explore new frontiers in this field.

**Methods:**

We reviewed the literature in the Web of Science Core Collection database from January 2000 to August 2022 using the retrieval strategy of medical subject headings combined with text words. We focused on articles and reviews that were published in English. Relevant data of the target publications, such as title, authors, organizations, abstract, keywords, published date, journal, and number of citations, were collected. The R software with the “bibliometrix” and VOSviewer software was used to process and visualize the information.

**Results:**

Among a total of 20,581 records related to HCM, 13,427 from 103 countries and regions, 8,676 affiliations, and 46,645 researchers were included. Most of the publications in this field were from the United States, followed by Japan, the United Kingdom, and China. We also report the top 10 institutions and most influential researchers, cited articles, and highest-frequency keywords (echocardiography, heart failure, sudden cardiac death, genetics, atrial fibrillation, magnetic resonance imaging/cardiac magnetic resonance, prognosis, mutation, arrhythmia, late gadolinium enhancement). In addition, keywords trend analysis indicated that the novel medicine Mavacamten, genetic diagnosis, and cardiac magnetic resonance have attracted the most attention for the treatment and diagnosis of HCM over the past five years.

**Conclusion:**

The present study reports on the global research trends of HCM over the past two decades using bibliometric analysis. It may enlighten new frontiers in the diagnosis, treatment, and risk prevention of HCM.

## 1. Introduction

Hypertrophic cardiomyopathy (HCM) is a primary type of heritable cardiomyopathy characterized by spontaneous genetic mutations and progressive myocardial hypertrophy. According to epidemiological investigations, the prevalence of HCM is approximately 1 per 200 in America and 800 per million in China. It is considered the main cause of sudden cardiac death in young people and even athletes (1–3). The current mainstream opinion regards mutations in genes encoding proteins of the cardiomyocytic contractile apparatus as the major pathogenesis of HCM. Nevertheless, obvious clinical heterogeneity in patients with HCM and the increasing number of clinical trials indicates certain limitations to the single gene mutation theory (4). With the development of high-throughput sequencing technology and bioinformatics, a more precise type of medication based on genomics, transcriptomes, proteomes, and metabolomes has made essential contributions to deepening the understanding of the genetic molecular mechanism underlying HCM (5, 6). With efforts such as the popularization of genetic diagnosis, accelerated research process of targeted therapeutic drugs (7), application of innovative operative treatment [Liwen Procedure (8)], employment of sudden cardiac death risk prediction models (9) and upgrade of management guidelines for HCM in 2020 (10), there have also been remarkable achievements in the diagnosis, treatment, and risk prevention of HCM over the past two decades.

**FIGURE 1:**
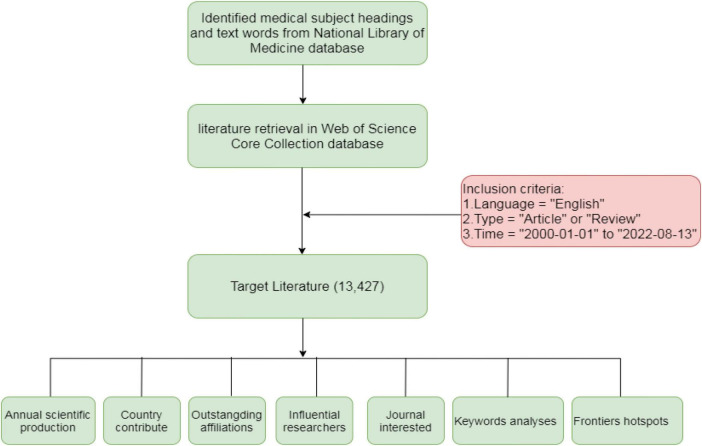
The flowchart of the research.

Given the rapid increase in the number of scientific publications on HCM, it is necessary to understand the change in research topics in this field over time and explore possible future trends. However, it remains a challenge to gain a comprehensive overview of the prominent research on HCM without the use of bibliometrics. Bibliometrics refers to the quantitative analysis of published academic literature using mathematical and statistical methods to explore the development of a certain research field within a specific time frame. It emphasizes the impact of publications; the contributions of individuals, institutions, and countries; as well as the main directions of future research (11). To the best of our knowledge, there is yet to be a bibliometric analysis on HCM. Thus, in this study, we conducted such an analysis to identify the global research trends on HCM over the past two decades and explore new frontiers in this topic. The flowchart of this research is illustrated in Figure 1.

## 2. Materials and methods

### 2.1. Literature source and retrieval strategy

This study followed the Strengthening the Reporting of Observational Studies in Epidemiology (STROBE) reporting guideline (12). Web of Science (WOS) is considered as a prominent multidisciplinary literature databases in the world, widely used to source data for bibliometric (13, 14). We reviewed the literature in the WOS Core Collection database from January 2000 to August 2022 using a retrieval strategy guided by medical subject headings combined with text words. The medical subject headings and text words were identified from the database of National Library of Medicine (https://www.ncbi.nlm.nih.gov/). The detailed retrieval strategy was set as follows: Subject term: [Hypertrophic Cardiomyopathy] or [Hypertrophic Obstructive Cardiomyopathies]; Type of literature: [articles and reviews]; Time span: [from 2000 to 2022]; Language selection: [English], Citation indexes: [SCI-Expanded] (15). Literature retrieval was conducted independently by two researchers (Xifeng Zheng and Zhen Jia). Relevant details of the target publications, such as title, authors, organizations, abstract, keywords, published date, published journal, references and number of citations were saved in “.txt” format.

### 2.2. Bibliometric analysis

We comprehensively analyzed the countries and regions, institutions, and researchers involved in HCM over the past two decades and report the top 10 contributing countries, outstanding institutions, influential researchers and the related structure of the international cooperation network in terms of involved nations, institutions, and researchers. Moreover, journals with most HCM-related publications, and the most cited articles of on HCM were also investigated. Finally, we explored the highest-frequency keywords and the hotspot changes in hotspots in this field using keyword trend analysis, with a standard filter setting of keywords frequency greater than 15 and more than three keywords per year. The R software (version 4.0.3) on the Biblioshiny app equipped with the “bibliometrix”program (16) (version 4.0), VOSviewer (version 1.6.18) (17) and Microsoft Excel 2016 were used to process and visualize data.

## 3. Results

### 3.1. Overview of the dataset

**FIGURE 2:**
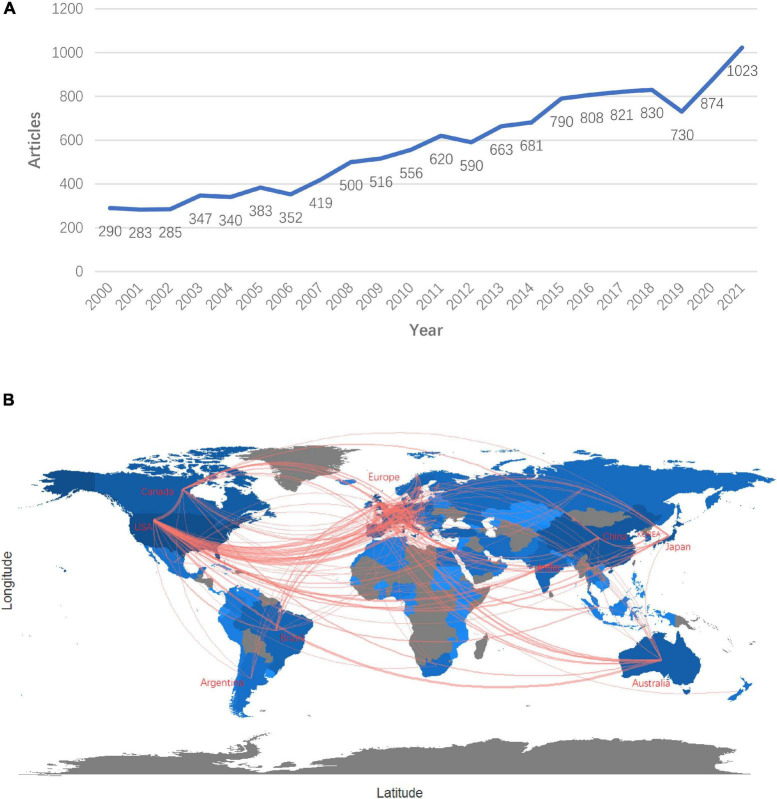
Panel **(A)** annual scientific productions since 2000. The abscissa represents time (years), and the ordinate represents the global number of articles on HCM published each year. Panel **(B)** major international cooperation distribution worldwide. The horizontal axis represents latitude, and the vertical axis represents longitude. The major countries and world regions advocating international hypertrophic cardiomyopathy research cooperation have been marked on the map.

Overall, 20,581 records related to HCM were obtained from the WOS database, and a total of 13,427 articles or literature reviews were selected for further analysis. In total, 103 countries and regions, 8,676 affiliations, and 46,645 researchers are involved in this field and the number of scientific publications is increasing year-on-year, with an annual growth rate of 3.44% (Figure 2A). International cooperation programs are mostly concentrated in America, Argentina, Australia, Brazil, Canada, China, European, India, Japan and Korea (Figure 2B, sorted by country initials).

### 3.2. Information on the top 10 countries, institutions, and scholars contributing to HCM research

**FIGURE 3:**
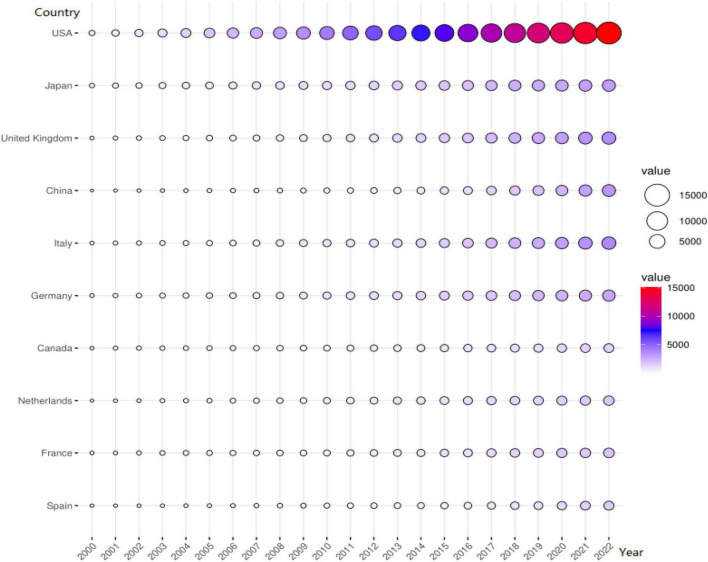
Scientific publication from the top 10 contributing countries over time. The abscissa represents time (years). The ordinate represents the top 10 countries with their cumulative scientific publications and related value over time (2000–2022).

**FIGURE 4:**
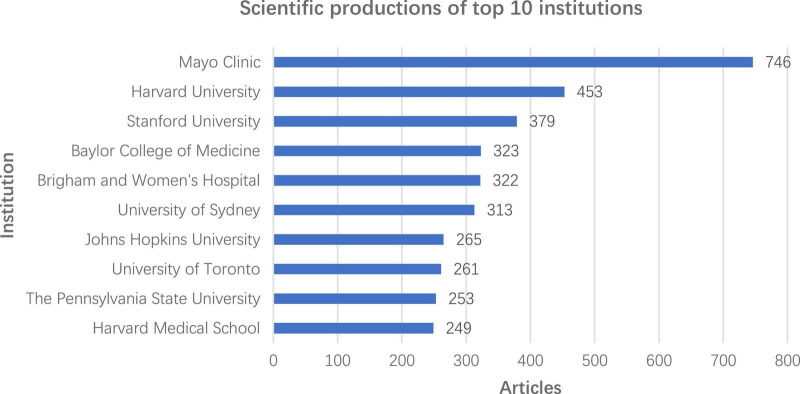
Scientific productions of top 10 institutions. The abscissa represents the total number of scientific productions since 2000, and the ordinate represents the top 10 institutions with the most accumulate scientific publications since 2000.

**FIGURE 5:**
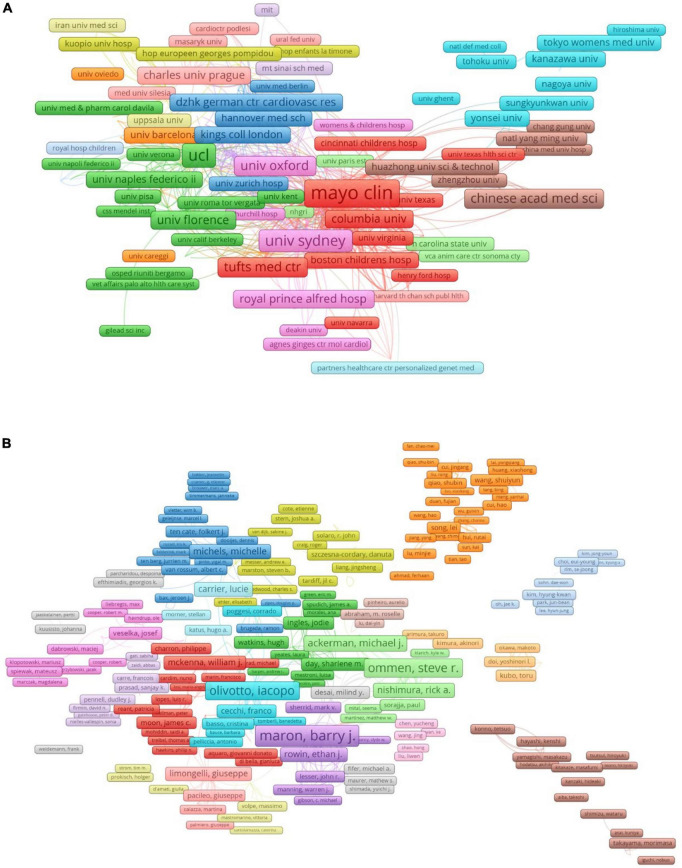
Cooperation network of worldwide outstanding institutions and researchers involved in HCM research. Panel **(A)** the cooperation network of worldwild outstanding affiliations. Panel **(B)** the cooperation network of worldwild outstanding researchers.

Globally, the United States accumulating contributed the most scientific publications on HCM, followed by Japan, the United Kingdom, China, Italy, Germany, Canada, the Netherlands, France, and Spain (Figure 3). The top 10 outstanding institutions are, in order, Mayo Clinic, Harvard University, Stanford University, Baylor College of Medicine, Brigham and Women’s Hospital, University of Sydney, Johns Hopkins University, University of Toronto, The Pennsylvania State University, and Harvard Medical School (Figure 4). The H-index represented an indicator to reflects a scholar’s academic achievement. It combines two key metrics, publication and citation counts, and is defined as the number of papers with citation number ≥ H (18, 19). The most influential scholar of HCM was Barry J Maron from Tufts Medical Center Hypertrophic Cardiomyopathy Center according to the H-index evaluation criteria, followed by William J McKenna (University College London), Martin S Maron (Tufts Medical Center Hypertrophic Cardiomyopathy Center), Michael J. Ackerman, Steve R. Ommen, Christine E. Seidman, Perry M. Elliott, Jonathan G. Seidman, Iacopo Olivotto, Jeffrey A. Towbin (Table 1). The cooperation networks of worldwide outstanding institutions and scholars were illustrated in Figure 5.

**TABLE 1:** The H-index of the top 10 influential scholars on HCM.

Scholar	H-index	Institution	Country
Barry J. Maron	93	Tufts Medical Center	USA
William J. McKenna	65	University College London	UK
Martin S. Maron	61	Tufts Medical Center	USA
Michael J. Ackerman	59	Mayo Clinic	USA
Steve R. Ommen	58	Mayo Clinic	USA
Christine E. Seidman	58	Harvard Medical School	USA
Perry M. Elliott	55	University College London	UK
Jonathan G. Seidman	53	Harvard Medical School	USA
Iacopo Olivotto	50	University of Florence	Italy
Jeffrey A. Towbin	47	Cincinnati Children’s Hospital Medical Center	USA

### 3.3. Influential journals and high-cited articles in the HCM research field

The top 10 journals with the most publications on HCM are, in order, the American Journal of Cardiology, Journal of the American College of Cardiology, Circulation, International Journal of Cardiology, Heart, Journal of the American Society of Echocardiography, Echocardiography, Circulation Journal, Journal of Molecular and Cellular Cardiology and European Heart Journal (Table 2). The top 10 most cited articles since year 2000 year are displayed in Table 3, most of which belong to are guidelines or literature reviews with significance in a certain stage.

**TABLE 2:** The top 10 journals with the most publications on HCM since 2000.

Journal	Article	IF (2022)	JCR (2022)	Publisher
American Journal of Cardiology	347	3.133	Q3	Elsevier
Journal of the American College of Cardiology	271	27.203	Q1	Elsevier
Circulation	237	39.918	Q1	Lippincott Williams and Wilkins
International Journal of Cardiology	227	4.039	Q2	Elsevier
Heart	192	7.365	Q1	BMJ Publishing Group
Journal of the American Society of Echocardiography	174	7.722	Q1	Elsevier
Echocardiography*	172	1.874	Q4	Wiley
Circulation Journal	155	3.350	Q3	Japanese Circulation Society
Journal of Molecular and Cellular Cardiology	155	5.763	Q2	Elsevier
European Heart Journal	144	35.855	Q1	Oxford University Press

*full name “Echocardiography: A Journal of Cardiovascular Ultrasound and Allied Techniques”.

IF, impact factor; JCR, Journal Citation Reports [Clarivate, 2022].

**TABLE 3:** Top 10 most cited articles on HCM since year 2000.

Authors	Title	Journal	Local citations	Global citations
Perry M. Elliott et al.	2014 ESC Guidelines on diagnosis and management of hypertrophic cardiomyopathy: The Task Force for the Diagnosis and Management of Hypertrophic Cardiomyopathy of the European Society of Cardiology (ESC) (41)	European Heart Journal	1,670	2,354
Barry J. Maron et al.	Hypertrophic cardiomyopathy: a systematic review (42)	Journal of the American Medical Association	1,212	1,550
Martin S. Maron et al.	Effect of left ventricular outflow tract obstruction on clinical outcome in hypertrophic cardiomyopathy (43)	New England Journal of Medicine	713	887
Pascale Richard et al.	Hypertrophic cardiomyopathy: distribution of disease genes, spectrum of mutations, and implications for a molecular diagnosis strategy (44)	Circulation	697	861
Bernard J. Gersh et al.	2011 ACCF/AHA Guideline for the Diagnosis and Treatment of Hypertrophic Cardiomyopathy: a report of the American College of Cardiology Foundation/American Heart Association Task Force on Practice Guidelines Developed in collaboration with the American Association for Thoracic Surgery, American Society of Echocardiography, American Society of Nuclear Cardiology, Heart Failure Society of America, Heart Rhythm Society, Society for Cardiovascular Angiography and Interventions, and Society of Thoracic Surgeons (45)	Journal of the American College of Cardiology	529	699
P Spirito et al.	Magnitude of left ventricular hypertrophy and risk of sudden death in hypertrophic cardiomyopathy (46)	New England Journal of Medicine	524	710
Barry J. Maron et al.	Clinical course of hypertrophic cardiomyopathy with survival to advanced age (47)	Journal of the American College of Cardiology	509	676
Perry Elliott et al.	Classification of the cardiomyopathies: a position statement from the European Society of Cardiology Working Group on Myocardial and Pericardial Diseases (48)	European Heart Journal	503	1592
Barry J. Maron et al.	Hypertrophic cardiomyopathy (49)	Lancet	502	639
Perry M. Elliott et al.	Sudden death in hypertrophic cardiomyopathy: identification of high-risk patients (50)	Journal of the American College of Cardiology	469	608

### 3.4. Keywords and hotspots trend analyses

**FIGURE 6:**
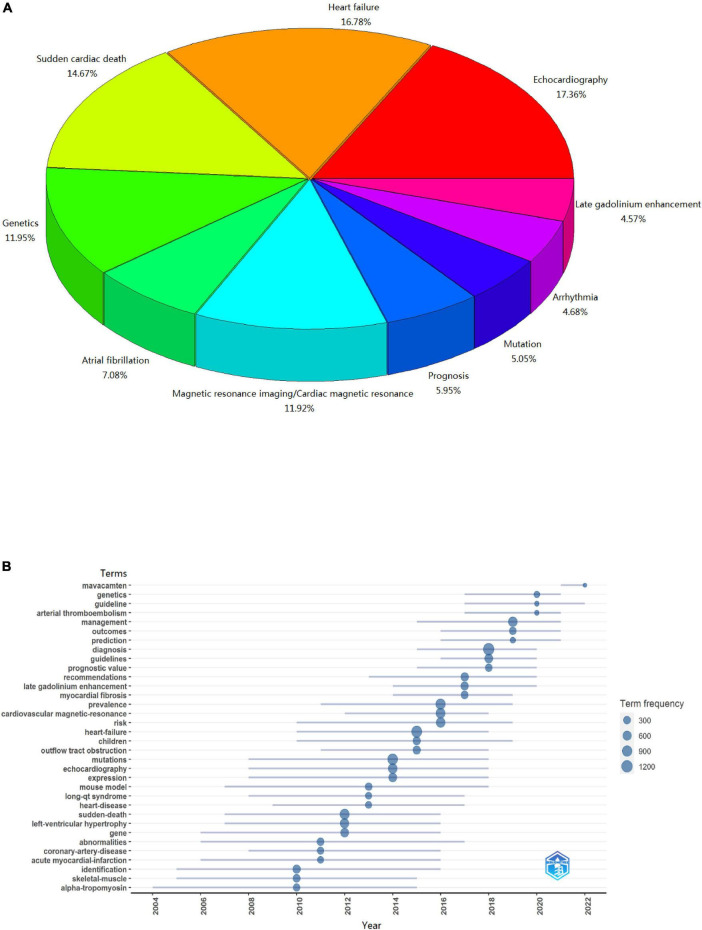
Panel **(A)** top 10 highest-frequency keywords about hypertrophic cardiomyopathy since year 2000. The figure shows the top 10 highest-frequency keywords and related percentages. Panel **(B)** keywords trend analyses results about hypertrophic cardiomyopathy since year 2010. The figure shows the annual top 3 highest-frequency keywords since 2010.

A total of 13,574 keywords were noted by the author as mentioned in the dataset. We identified echocardiography, heart failure, sudden cardiac death, genetics, atrial fibrillation, magnetic resonance imaging/cardiac magnetic resonance, prognosis, mutation, arrhythmia, and late gadolinium enhancement as the top 10 keywords. (Figure 6A). These keywords can be categorized into two topics focusing on diagnosis methods and clinical complications of HCM. In addition, we summarized the keyword trends from 2010 to 2022 and explored the hotspots in HCM. The results indicate that the novel medicine Mavacamten, genetic tests, and cardiac magnetic resonance attracted the most attention in the treatment and diagnosis of HCM in recent years (Figure 6B).

## 4. Discussion

In this present study, quantitative statistical analysis of the scientific literature in the field of HCM over the past 2 decades was conducted using bibliometrics. The results showed that the number of papers in this field is increasing year-on-year, with an annual growth rate of 3.44%. This indicates that clinical researchers are maintaining a continuous interest in this field. With gradually strengthening international cooperation, our knowledge in this field is also constantly improving. The United States plays a leading role in this field, which is reflected by its many outstanding scientific research institutions and influential researchers, international cooperation initiatives, and the largest number of influential scientific achievements. Through the analysis of the development trends, we found that the research interest in this field focuses mainly on diagnostic technology (such as genetic testing and cardiac imaging examination), risk of major adverse cardiac events prevention and prognosis evaluation, and novel treatment methods.

Echocardiography, magnetic resonance imaging, cardiac magnetic resonance (CMR), and late gadolinium enhancement (LGE) account for four of the top 10 most frequent keywords, indicating that cardiac imaging is essential to the diagnosis of HCM. Echocardiography is the first-choice imaging method in HCM evaluation and family screening owing to its non-invasiveness and convenience. Compared with echocardiography, CMR not only possesses an advantage in terms of image quality, but it is also more suited for risk stratification and prognostic prediction (20). Specifically, CMR imaging combined with LGE can directly reflect the degree of myocardial fibrosis in HCM and has become a research hotspot in recent years (21). Myocardial fibrosis is a common pathological state in HCM and various other cardiac diseases and is an essential cause of ventricular tachyarrhythmia caused by reentrant activity and initiation trigger mechanisms (22). Multiple clinical studies and meta-analyses have shown that ventricular fibrosis detected using LGE is a powerful predictor of sudden cardiac death (SCD) events in patients with HCM (21, 23–26). The advanced cardiac imaging functional assessment combined with the SCD risk prediction model based on traditional clinical parameters (9) provides individualized assessment for the risk stratification of the HCM population including SCD events risk.

Genetic testing, followed by the rapid evolution of next-generation sequencing, has become more common and significant in identifying asymptomatic HCM patients with positive genetic types and differential diagnosis with other inherited heart diseases (such as Fabry disease (27) or hereditary transthyretin amyloidosis) that may manifest with similar phenocopies. In addition, genetic testing can assist in the diagnosis of population with family history and guide treatment management of relatives potentially at risk to prevent adverse outcomes (28). Thus, genetic testing has been recommended by the 2020 AHA/ACC guideline of HCM as a prominent diagnostic procedure during family screening (10).

Medication and surgery/interventional therapy are the two major forms of treatment for HCM. Medication includes beta-receptor blockers, which are suitable for both patients with obstructive and non-obstructive HCM, however, efficacy may be limited for patients with severely obstructive symptoms (29). A small-molecule inhibitor of sarcomere contractility named MYK-461 has attracted the attention of the academic community since 2016 because it can reduce contractility by decreasing the adenosine triphosphatase activity of the cardiac myosin heavy chain and suppress the development of ventricular hypertrophy, cardiomyocyte disarray, and myocardial fibrosis in mice (7). Subsequently, the novel medication Mavacamten originated from MYK-461 has achieved encouraging results in several clinical trials of patients with hypertrophic obstructive cardiomyopathy manifesting as improved post-exercise left ventricular outflow tract pressure gradients, exercise tolerance, and clinical symptoms (30–34). Thus, Mavacamten received its first approval on April 28, 2022 in the United States for the treatment of adults with symptomatic New York Heart Association (NYHA) class II–III obstructive HCM, to improve functional capacity and symptoms (35). However, evidence from clinical trials on the treatment of non-obstructive hypertrophic cardiomyopathy with Mavacamten is currently lacking, which is a direction of concern in the future.

The surgical and interventional therapy methods consist of septal myectomy (SM), alcohol septal ablation (ASA), and pacemaker treatment. SM therapy was first proposed by Morrow in 1961 and improved by Messmer in 1994. It has a definite curative effect in clinical practice and is considered the gold-standard treatment for medically refractory obstructive HCM. For elderly patients with chronic diseases who cannot tolerate SM operation, the ASA procedure is a relatively effective and safe alteration (36, 37). Besides the traditional operations mentioned above, it is worth noting that an innovative type of surgery named the Liwen procedure has been proposed by Chinese clinicians in recent years. This new process is echocardiography-guided and involves a myocardial biopsy and percutaneous intramyocardial septal radiofrequency ablation, which is remarkably effective in practice. This procedure may revolutionize the treatment of obstructive HCM (8, 38–40).

Compared with traditional literature reviews, a bibliometric analysis can provide better insight into the evolving research on relevant foci and trends via comprehensive and objective data analysis. Our analysis is exclusively based on all objective information, without supervision interferences which may have led to further bias. Nonetheless, the study has some limitations. First, our dataset was retrieved from the WOS database alone. Although the WOS database has been widely used for bibliometric studies because of the recording of the citation information of the literature, which counts for a vital part of research output indicators, some articles might still have been missed. Second, the study only focused on original research and literature reviews published in English; therefore, selection bias was inevitable.

## 5. Conclusion

The present study reports on the global research trends of HCM over the past 2 decades using bibliometric analysis. It may enlighten new frontiers in the diagnosis, treatment, and risk prevention of HCM.

## Data availability statement

The original contributions presented in this study are included in the article/supplementary material, further inquiries can be directed to the corresponding author.

## Ethics statement

Ethical review and approval was not required for this study in accordance with the local legislation and institutional requirements.

## Author contributions

XZ was involved in the conception and manuscript writing. ZJ was responsible for conception and scientific supervision. ZH and ML were involved in statistics and software. All authors reviewed and approved the final manuscript.
